# Caractéristiques et facteurs prédictifs de décès dans les syndromes coronariens aigus du sujet âgé: données du Registre des syndromes coronariens aigus de l'Institut de cardiologie d'Abidjan

**DOI:** 10.48327/mtsi.v3i1.2023.269

**Published:** 2023-02-22

**Authors:** Hermann Yao, Esther Ehouman, Didier Kouadio, Camille Touré, Elvis Sepih, Isabelle Kouamé, Arnaud Ekou, Roland N'guetta

**Affiliations:** Institut de cardiologie d'Abidjan, 01 BP V 206 Abidjan, Côte d'Ivoire

**Keywords:** Syndrome coronarien aigu, Sujet âgé, Abidjan, Côte d'Ivoire, Afrique subsaharienne, Acute coronary syndrome, Elderly, Abidjan, Côte d'Ivoire, Sub-Saharan Africa

## Abstract

**Introduction-Objectif:**

Les syndromes coronariens aigus (SCA) représentent la première cause de décès chez le sujet âgé en Afrique subsaharienne. Le but de cette étude était d'analyser les caractéristiques des SCA chez le sujet âgé à l'Institut de cardiologie d'Abidjan.

**Matériels et méthodes:**

Étude transversale du 1^er^ janvier 2015 au 31 décembre 2019. Tous les patients d'au moins 18 ans, admis à l'Institut de cardiologie d'Abidjan pour SCA ont été inclus. Ces patients ont été divisés en deux groupes: patients âgés (≥ 65 ans) et moins âgés (< 65 ans). Les caractéristiques cliniques, le traitement reçu et l’évolution des deux groupes ont été comparés et analysés.

**Résultats:**

Au total, 570 patients ont été inclus, dont 137 (24%) avaient plus de 65 ans. Soixante pour cent (60%) des patients âgés ont présenté un SCA avec sus-décalage persistant du segment ST (SCA ST+). L'angioplastie artérielle transluminale était peu réalisée chez les patients âgés (21,1% *vs* 30,2%, p = 0,039). L'insuffisance cardiaque était la complication la plus importante dans le groupe des patients âgés (56,9% vs 44,6%, p = 0,012). La mortalité hospitalière était de 8% chez les patients âgés. Les facteurs associés à la mortalité hospitalière étaient l'antécédent d'hypertension artérielle (OR 2,58; IC95% 1,10-6,08) et la forme SCA ST+ (OR 11,60; IC95% 2,70-49,76). La réalisation d'une angioplastie artérielle transluminale était un facteur protecteur de la mortalité intra-hospitalière (OR 0,14; IC95% 0,03-0,62).

**Conclusion:**

Les syndromes coronariens aigus surviennent à une fréquence croissante avec l’âge. La gravité du pronostic chez le sujet âgé est sous-tendue par la forme clinique et les comorbidités. L'angioplastie coronaire réduit considérablement la mortalité hospitalière.

## Abstract (en)

**Introduction-Objective:**

Acute coronary syndromes (ACS) are the leading cause of death among the elderly in sub-Saharan Africa. The aim of this study was to analyze the characteristics of ACS among the elderly at the Abidjan Heart Institute.

**Materials and methods:**

Cross-sectional study from January 1, 2015, to December 31, 2019. All patients aged 18 or more admitted to the Abidjan Heart Institute for ACS were included. These patients were divided into two groups: elderly (≥ 65 years old) and non-elderly (< 65 years old). Clinical data, management and outcomes were compared and analyzed in both groups.

**Results:**

A total of 570 patients were included, of which 137 (24%) were elderly. Sixty percent (60%) of elderly patients presented with ST Segment Elevation Myocardial Infarction (STEMI). Percutaneous coronary intervention (PCI) was less performed among elderly patients (21.1% vs 30.2%, p = 0.039). Heart failure was the most important complication among the elderly group (56.9% vs 44.6%, p = 0.012). In-hospital mortality was 8% among the elderly. Predictive factors for in-hospital mortality were history of hypertension (HR 2.58; CI95% 1.10-6.08) and STEMI presentation (OR 11.60; CI95% 2.70-49.76). PCI was a protective factor for in-hospital mortality (OR 0.14; IC95% 0.03-0.62).

**Conclusion:**

ACS occur with increasing frequency with age. Poor outcomes among the elderly are determined by the clinical presentation and comorbidities. PCI appears to significantly reduce in-hospital mortality.

## Introduction

L'Afrique subsaharienne connaît une transition épidémiologique vers les maladies non transmissibles, et en particulier les maladies cardiovasculaires à cause de l'urbanisation rapide, l'occidentalisation du mode de vie, la prévalence croissante des facteurs de risque cardiovasculaire, l'amélioration de la qualité des soins de santé et le vieillissement de la population [19, 23]. Dans cette région, la mortalité par maladies cardiovasculaires représente environ 14% de la mortalité générale; la proportion la plus importante étant attribuée à la maladie coronaire [15]. En Afrique subsaharienne, les syndromes coronariens aigus (SCA), forme la plus grave de la maladie coronaire, sont en hausse. Ils représentent 22,3% des hospitalisations en cardiologie en Côte d'Ivoire [21]. Bien que touchant le plus souvent le sujet jeune, les SCA représentent la première cause de décès chez le sujet âgé dans nos régions [15]. En effet les syndromes coronariens aigus chez les patients âgés sont graves, avec une présentation clinique d'autant moins typique et un pronostic d'autant plus sombre qu'ils surviennent chez des patients polypathologiques et vulnérables [16]. Il n'existe pas de recommandations claires et spécifiques sur la stratégie optimale de prise en charge des SCA chez le sujet âgé [12]. Toutefois l'angioplastie coronaire reste une stratégie thérapeutique de référence chez des patients pris en charge précocement [4]. Peu d’études se sont intéressées aux SCA du sujet âgé en Afrique subsaharienne. En Côte d'Ivoire plusieurs études sur les SCA ont été réalisées, mais aucune ne s'est intéressée au sujet âgé. Par conséquent, la présente étude avait pour but de déterminer les caractéristiques des SCA chez le sujet de plus de 65 ans et d'identifier les facteurs pronostiques des SCA à l'Institut de cardiologie d'Abidjan (ICA).

## Matériels Et Méthodes

### Critères de sélection

Nous avons réalisé une étude observationnelle transversale, allant du 1^er^ janvier 2015 au 31 décembre 2019, à partir du registre des SCA de l'ICA, centre national de référence des maladies cardiovasculaires en Côte d'Ivoire.

Ont été inclus de façon anonyme tous les patients âgés d'au moins 18 ans, admis à l'ICA pour SCA au cours de la période d’étude. Les patients pour lesquels les données étaient indisponibles ou incomplètes n'ont pas été inclus. Le diagnostic de SCA était retenu devant une symptomatologie évocatrice d'ischémie myocardique, avec ou sans sus-décalage du segment ST sur l’électrocardiogramme (ECG), accompagnée ou non d'une élévation des marqueurs biologiques de nécrose myocardique. Ces critères diagnostiques ont été établis conformément au code I21.9 de la classification internationale des maladies, 10^e^ révision (CIM-10) [7]; et à la définition universelle de l'infarctus du myocarde en vigueur [17, 18]. À partir de cette population, deux groupes ont été définis et comparés en fonction de l’âge: le premier groupe constitué de patients de moins de 65 ans et le second constitué de patients de 65 ans et plus.

### Paramètres étudiés

Les paramètres étudiés concernaient les données sociodémographiques et anthropométriques, les antécédents et facteurs de risque de la maladie coronaire, le délai de prise en charge, les caractéristiques cliniques, les données de l'ECG et de l'imagerie, les caractéristiques biologiques, les complications hospitalières et la prise en charge.

### Analyse statistique

Les variables qualitatives ont été exprimées sous forme d'effectifs et de pourcentages; les variables quantitatives ont été exprimées sous forme de médiane et d'intervalle interquartile Les variables qualitatives ont été comparées à l'aide du test du Khi-carré de Pearson ou du test exact de Fisher. Les variables quantitatives ont été comparées à l'aide du test de Student ou celui de Wilcoxon. Des analyses univariée et multivariée par un modèle de régression logistique ont été réalisées pour déterminer les facteurs associés à la mortalité hospitalière, avec un seuil d'inclusion de p ≤ 0,10 dans l'analyse multivariée. Les variables dépendantes ont été incluses dans le modèle univarié sur la base des données connues de la littérature. Le test du rapport de vraisemblance a été utilisé pour évaluer la significativité des Odds Ratios (OR) et de leurs intervalles de confiance à 95%. Le seuil de significativité retenu pour les tests statistiques était fixé à 5%. L'analyse statistique a été réalisée à l'aide du logiciel SPSS version 25 (IBM, Armonk, NY).

## Résultats

Au total 570 patients ont été inclus dans l'analyse, parmi lesquels 433 (76%) avaient moins de 65 ans et 137 (24%) avaient plus de 65 ans. La population étudiée était majoritairement masculine. Toutefois, il y avait une proportion plus élevée de femmes atteintes de SCA chez les sujets âgés (34,3% contre 14,3%, p < 0,001). Les caractéristiques de base selon la catégorie d’âge sont comparées dans le Tableau I. Le diabète (37,2% contre 24%, p = 0,002) et l'hypertension artérielle (HTA) (73,7% contre 50,8%, p < 0,001) étaient les facteurs de risque cardiovasculaire les plus importants chez les sujets âgés comparativement aux sujets jeunes. Il n'y avait pas de différence statistique significative concernant le type de douleur entre les deux groupes. On notait une proportion plus importante de sujets âgés présentant une insuffisance ventriculaire gauche selon la classification de Killip (37,2% contre 22,2%, p < 0,001). À l’échocardiographie, l'altération de la fraction d’éjection ventriculaire gauche était également plus fréquente chez les sujets âgés (17,5% contre 10,9%, p = 0,04). Le débit de filtration glomérulaire chez les sujets âgés était significativement plus bas (52,0 [32,8-74,8] contre 85,2 [64,7-102,9], p < 0,001). Il n'y avait pas de différence statistique significative concernant le diagnostic final de SCA.

**Tableau I:** Caractéristiques générales de la population d’étude (n(%) ou médiane [intervalle interquartiles]) General characteristics of the study population (number and proportion or median and interquartile range)

	Moins de 65 ans n = 433	65 ans et plus n = 137	Valeur de p
**Facteurs de risque et antécédents cardiovasculaires**			
sexe féminin	62 (14,3)	47 (34,3)	< 0,001
hypertension artérielle	220 (50,8)	101 (73,7)	< 0,001
diabète	104 (24,0)	51 (37,2)	0,002
dyslipidémie	124 (28,6)	37 (27,0)	0,712
tabagisme actif	113 (26,1)	12 (8,8)	< 0,001
obésité	118 (27,3)	29 (21,2)	0,156
antécédents d'AOMI	7 (1,6)	1 (0,7)	0,442
antécédents d'AVC	17 (3,9)	11 (8,0)	0,052
antécédents de coronaropathie	121 (27,9)	21 (15,3)	0,220
**Clinique**			
délai admission < 12 h	197 (45,5)	60 (43,8)	0,727
douleur typique	271 (62,7)	84 (61,3)	0,789
stade Killip ≥ 2	96 (22,2)	51 (37,2)	< 0,001
FEVG < 40%	47 (10,9)	24 (17,5)	0,040
Diagnostic			
SCA ST+	270 (62,4)	82 (59,9)	0,472
SCA ST-Troponine+	81 (18,7)	32 (23,3)	0,275
SCA ST-Troponine-	82 (18,9)	23 (16,8)	
Biologie			
pic glycémie, g/l	1,26 [1,02 - 1,77]	1,47 [1,13 - 2,22]	0,034
hémoglobine, g/dl	14,10 [13,00 - 15,20]	13,00 [11,82 - 14,37]	< 0,001
DFG, ml/min/1,73 m^2^	85,22 [64,70 - 102,90]	52,00 [32,80 - 74,85]	< 0,001

AOMI: artériopathie oblitérante des membres inférieurs. AVC: accident vasculaire cérébral. FEVG: fraction d’éjection du ventricule gauche. SCA ST+: syndrome coronarien aigu avec sus-décalage persistant du segment ST. SCA ST- T+: syndrome coronarien aigu sans sus-décalage persistant du segment ST à troponine I positive. SCA ST- T-: syndrome coronarien aigu sans sus-décalage persistant du segment ST à troponine I négative. DFG: débit de filtration glomérulaire.

Le Tableau II compare la stratégie de reperfusion (invasive ou non invasive) utilisée et les traitements adjuvants administrés chez les sujets jeunes et les sujets âgés. L'angioplastie coronaire transluminale était moins réalisée chez les sujets âgés (30,2% contre 21,2%, p = 0,039). Les inhibiteurs de l'enzyme de conversion (p = 0,040) étaient peu utilisés chez les sujets âgés. En revanche le furosémide était plus utilisé chez les sujets âgés (p = 0,001).

**Tableau II:** Prise en charge hospitalière (Abidjan, 2015-2019) (n(%)) In-hospital management (Abidjan, 2015-2019) (n(%))

	Moins de 65 ans n = 433	65 ans et plus n = 137	Valeur de p
Thrombolyse	57 (13,2)	4 (2,9)	0,001
Angioplastie	131 (30,2)	29 (21,2)	0,039
HBPM	262 (60,5)	80 (58,4)	0,660
HNF	77 (17,8)	26 (19,0)	0,751
Aspirine	382 (88,2)	115 (83,9)	0,191
Clopidogrel	392 (90,5)	119 (86,9)	0,219
Bêtabloquants	340 (78,5)	100 (73,0)	0,179
Statines	375 (86,6)	120 (87,6)	0,766
IEC	329 (76,0)	92 (67,2)	0,040
Furosémide	126 (29,1)	60 (43,8)	0,001
Dérivés nitrés	60 (13,9)	22 (16,1)	0,522
Anticalcique	42 (9,7)	12 (8,8)	0,743

HNF: héparine non fractionnée. HBPM: héparine de bas poids moléculaire. IEC: inhibiteur de l'enzyme de conversion.

Les complications en cours d'hospitalisation selon la catégorie d’âge sont présentées dans le Tableau III. Plus de la moitié des sujets âgés ont présenté une insuffisance ventriculaire gauche en cours d'hospitalisation, cette proportion étant plus élevée que celle des sujets jeunes avec une différence statistiquement significative (56,9% contre 44,6%, p = 0,012). Il existait une tendance, quoique non significative, à une proportion plus importante de décès chez les sujets âgés (8% contre 4,8%, p = 0,159).

**Tableau III:** Complications en cours d'hospitalisation (Abidjan, 2015-2019) (n(%)) In-hospital complications (Abidjan, 2015-2019) (n(%))

	Moins de 65 ans n = 433	65 ans et plus n = 137	Valeur de p
Complications mécaniques	4 (0,9)	0 (0,0)	0,259
Complications thromboemboliques	12 (2,8)	3 (2,2)	0,711
Choc cardiogénique	5 (1,2)	4 (2,9)	0,149
Réaction péricardique	12 (2,8)	5 (3,6)	0,598
Extension de la nécrose	46 (10,6)	10 (7,3)	0,255
Décès	21 (4,8)	11 (8,0)	0,159
Trouble de la conduction	42 (9,7)	23 (16,8)	0,023
Trouble du rythme	100 (23,1)	36 (26,3)	0,446
Insuffisance ventriculaire gauche	193 (44,6)	78 (56,9)	0,012

Le Tableau IV montre l'analyse multivariée des facteurs associés à la mortalité hospitalière des deux groupes confondus. L’âge ≥ 65 ans n’était pas un facteur prédictif de mortalité hospitalière. Les prédicteurs de décès en cours d'hospitalisation étaient l'HTA (OR 2,58; IC95% 1,10-6,08) et le SCA avec sus-décalage persistant du segment ST (SCA ST+) (OR 11,60; IC95% 2,70-49,76). L'angioplastie artérielle transluminale était un facteur protecteur de décès en cours d'hospitalisation (OR 0,14; IC95% 0,03-0,62).

**Tableau IV:** Analyses univariée et multivariée des facteurs associés au décès hospitalier (Abidjan, 2015-2019) Univariate and multivariate analyses of predictors for in-hospital death (Abidjan, 2015-2019)

	Analyse univariée	Analyse multivariée		
**Variables**	**OR (IC 95%)**	**Valeur de p**	**OR (IC 95%)**	**Valeur de p**
Âge ≥ 65 ans	1,71 (0,80 – 3,649)	0,163	-	-
Sexe féminin	1,03 (0,41 – 2,56)	0,960	-	-
Tabagisme actif	0,35 (0,11 – 1,18)	0,090	-	-
Dyslipidémie	0,45 (0,17 – 1,20)	0,111	-	-
Diabète	1,23 (0,57 – 2,66)	0,596	-	-
Hypertension artérielle	2,43 (1,07 – 5,52)	0,033	2,58 (1,10 – 6,08)	0,030
Délai symptôme-admission	0,93 (0,45 – 1,89)	0,834	-	-
DFG, mL/min/1,73 m^2^	0,99 (0,97 – 1,00)	0,137	-	-
FEVG < 40%	2,51 (1,08 – 5,83)	0,032	1,58 (0,65 – 3,83)	0,314
SCA ST+	10,06 (2,38 – 42,54)	0,002	11,60 (2,70 – 49,76)	0,001
Angioplastie artérielle transluminale	0,16 (0,04 – 0,68)	0,013	0,14 (0,03 – 0,62)	0,009
Insuffisance ventriculaire gauche	1,27 (0,62 – 2,589)	0,516	-	-

DFG: débit de filtration glomérulaire. FEVG: fraction d'ejection du ventricule gauche. SCA ST+: syndrome coronarien aigu avec sus-décalage persistant du segment ST.

## Discussion

Depuis 1990, nous avons observé à Abidjan une augmentation progressive du nombre de patients atteints de SCA, qui serait associée à l'urbanisation rapide, à la progression des facteurs de risque cardiovasculaire, à la carence en médecine préventive et à la disponibilité d'outils diagnostiques performants (troponine, salle de cathétérisme) [9, 13, 21, 22].

La présente étude a montré que les SCA chez les sujets de plus de 65 ans sont fréquents à Abidjan avec une part importante de femmes. La présentation clinique était plus sévère et généralement associée à une altération de fonction rénale; ce qui entravait la mise en place d'une stratégie de prise en charge agressive chez des patients à haut risque cardiovasculaire dominé par l'HTA et le diabète.

Notre étude souffre cependant de certaines limites. En effet, le seuil choisi pour définir les patients âgés n'est pas uniforme dans la littérature. Aussi, le caractère monocentrique de l’étude a réduit le spectre de patients sélectionnés. De plus les données ont été recueillies de manière rétrospective, d'où le manque de données sur le niveau socio-économique mais aussi l'absence d’évaluation gériatrique standardisée de nos patients âgés qui devait nous permettre d'avoir des informations importantes, notamment sur l'ensemble des comorbidités et l’état de fragilité de ces patients. Ces éléments jouent un rôle important dans le pronostic du sujet âgé [1].

La proportion de femmes de plus de 65 ans atteintes de SCA de notre série était élevée. Ces résultats sont en concordance avec ceux de l’étude tunisienne de Ben Ahmed *et al.* où l'on observait une inversion de la prédominance masculine à partir de 80 ans [3]. Marie *et al.* au Sénégal retrouvaient un âge moyen de 68 ans dans leur série s'intéressant aux SCA chez les femmes [11]. Cela pourrait s'expliquer par l'augmentation du risque cardiovasculaire après la ménopause et l’évolution de l'espérance de vie entre les deux sexes.

L'hypertension artérielle représentait le facteur de risque cardiovasculaire le plus fréquent chez les sujets âgés de notre étude et était un facteur indépendant associé au risque de décès. La série tunisienne retrouvait un taux similaire d'HTA parmi les sujets âgés [3]. De plus, notre travail montrait une proportion importante du diabète chez les sujets de notre étude, qui s'expliquerait par la diminution de la masse musculaire au profit de la masse adipeuse, la répartition androïde de la masse graisseuse et la diminution de l'activité physique [6, 14].

Dans notre étude, pour plus de la moitié des sujets âgés le délai entre le début de leurs symptômes et leur admission à l'ICA était supérieur à 12 heures. Ce retard de prise en charge, observé également dans la littérature [8, 20], pourrait être lié au transit des patients dans un circuit pré-cardiologique très peu structuré dans notre contexte, mais aussi à une plus forte proportion de présentations cliniques atypiques particulièrement chez les patients âgés [5, 13]. Une attention particulière devrait être donc accordée chez les personnes âgées aux symptomatologies atypiques pour minimiser le retard diagnostique et améliorer le pronostic.

Les sujets âgés de notre étude avaient un faible débit de filtration glomérulaire, ce qui a limité la prise en charge à deux niveaux: la coronarographie nécessitant une injection de produit de contraste iodé pouvant aggraver l'insuffisance rénale, et la contre-indication de certaines thérapeutiques en cas d'insuffisance rénale. On notait une utilisation plus importante du furosémide chez les patients âgés car son efficacité est maintenue quel que soit l’âge, même en cas d'insuffisance rénale sévère.

Le sous-type SCA ST+ représentait le plus important facteur prédictif de décès en cours d'hospitalisation dans notre étude. Bahiru *et al.* au Kenya quant à eux montraient que les SCA ST+ présentaient le taux le plus élevé de mortalité en cours d'hospitalisation et d’évènements cardiovasculaires majeurs [2]. Par ailleurs, la réalisation de l'angioplastie dans notre série était associée à une réduction relative du risque de décès en cours d'hospitalisation de 86%. Nos travaux ont montré que l’âge ≥ 65 ans n’était pas associé à un risque de décès en cours d'hospitalisation comme la série kenyane [2]. Pourtant, le bénéfice estimé de la procédure invasive au cours de la phase aiguë du SCA est encore un sujet de débat chez les personnes âgées [10].

## Conclusion

Les SCA surviennent à une fréquence croissante avec l’âge de la population. La gravité du pronostic chez le sujet âgé est sous-tendue par les SCA ST+ et les comorbidités qui rendent compte de complications majeures accrues et d'une plus grande mortalité hospitalière. L'angioplastie coronaire représente un des moyens qui semble réduire considérablement cette mortalité. Par ailleurs, l'amélioration de la survie dans cette population suppose une meilleure identification par toutes les structures de soins et un transfert plus rapide en vue d'une revascularisation myocardique précoce. Dans notre contexte, les perspectives résident dans le développement de stratégies plus efficaces permettant la réduction de la progression de l'athérosclérose et de la maladie coronaire dans notre population potentiellement vieillissante.

## Contribution Des Auteurs

H. Yao, A. Ekou, R. N'guetta: conception de l’étude.

H. Yao, D. Kouadio: rédaction et correction du protocole et du manuscrit, réalisation de l'analyse statistique.

E. Ehouman, C. Touré, E. Sepih, D. Kouadio: collecte des données.

H. Yao, R. N'guetta: supervision, validation du manuscrit.

**LIENS D'INTÉRÊT.**

Les auteurs ne déclarent aucun lien d'intérêt
